# A Novel Approach to Vaccine Development: Concomitant Pathogen Inactivation and Host Immune Stimulation by Peroxynitrite

**DOI:** 10.3390/vaccines10101593

**Published:** 2022-09-22

**Authors:** Shahar Rotem, Erez Bar-Haim, Uri Elia, Hila Cohen, Shirley Lazar, Ofer Cohen, Theodor Chitlaru, Yoav Gal

**Affiliations:** Department of Molecular Genetics and Biochemistry, Israel Institute of Biological Research, Ness Ziona 74100, Israel

**Keywords:** reactive oxygen species, immunity, antigenicity

## Abstract

The design of efficient vaccines for long-term protective immunity against pathogens represents an objective of utmost public health priority. In general, live attenuated vaccines are considered to be more effective than inactivated pathogens, yet potentially more reactogenic. Accordingly, inactivation protocols which do not compromise the pathogen’s ability to elicit protective immunity are highly beneficial. One of the sentinel mechanisms of the host innate immune system relies on the production of reactive nitrogen intermediates (RNI), which efficiently inactivate pathogens. Peroxynitrite (PN) is a prevalent RNI, assembled spontaneously upon the interaction of nitric oxide (NO) with superoxide. PN exerts its bactericidal effect by via the efficient oxidation of a broad range of biological molecules. Furthermore, the interaction of PN with proteins results in structural/chemical modifications, such as the oxidation of tryptophan, tyrosine, and cysteine residues, as well as the formation of carbonyl, dityrosine, and nitrotyrosine (NT). In addition to their role in innate immunity, these PN-mediated modifications of pathogen components may also augment the antigenicity of pathogen peptides and proteins, hence contributing to specific humoral responses. In the study reported here, a novel approach for vaccine development, consisting of pathogen inactivation by PN, combined with increased immunity of NT-containing peptides, is implemented as a proof-of-concept for vaccination against the intracellular pathogen *Francisella tularensis* (*F. tularensis*). In vivo experiments in a murine model of tularemia confirm that PN-inactivated *F. tularensis* formulations may rapidly stimulate innate and adaptive immune cells, conferring efficient protection against a lethal challenge, superior to that elicited by bacteria inactivated by the widely used formalin treatment.

## 1. Introduction

Vaccination is the most effective medical intervention used to reduce death and morbidity caused by infectious diseases, commonly resulting in long-term protective immunity to bacterial and viral pathogens. In general, live attenuated vaccines exhibit superior efficacy compared to subunit and/or inactivated vaccines, due to their higher potential for recapitulating the interaction of the pathogen with the host in the course of infection, resulting in the elicitation of a variety of innate, humoral, and inflammatory responses [1,2]. However, this high efficiency needs to be accompanied by significant attenuation, which is essential for their safe use. The reactogenicity, which may be associated with these vaccines due to insufficient virulence attenuation, often precludes their large-scale human use [3]. Conversely, attenuation may compromise the ability of the pathogen to elicit a protective immune response due to the absence of essential antigens as a consequence of their virulence loss. Another type of vaccine, the subunit-based vaccine, usually relies on the administration of a limited (sometimes unique) number of antigens, and therefore fails to elicit immune responses against all potential protective epitopes exhibited by a given pathogen. Therefore, neutralized full pathogens, allowing presentation of a wide variety of protective antigens with minimal reactogenicity, represent an attractive alternative for whole-pathogen vaccine formulations, circumventing some of the above-mentioned limitations.

Formalin treatment is the most frequent modality for pathogen neutralization. However, cases of vaccine-enhanced disease and compromised antigenicity were attributed to the formalin-induced structural modification of antigens [4]. The negative effect on antigenicity may indicate the blocking of neutralizing epitopes in the formalin-inactivated pathogen, leading to the development of a non-protective antibody response [5]. Th2 responses were described following immunization with formalin-inactivated whole pathogens such as RSV and measles [4]. Beta-propiolactone treatment also served for inactivation purposes; however, as reported in the case of SARS-CoV-2 vaccines, this treatment exhibited limited efficiency [6].Thus, the development of improved methodologies for the inactivation of whole-pathogen vaccines represents an issue of high priority and accordingly; this is the subject of the study described in the current report.

The innate immune system consists of a plethora of mechanisms aimed at preventing or limiting pathogen colonization and expansion, including non-specific pathogen inactivation by reactive nitrogen intermediates (RNIs) [7]. RNIs are potent anti-microbials, superior to reactive oxygen species (ROS), owing to their longer half-lives and greater ability to penetrate membranes, enabling efficient pathogen elimination from vacuoles and cytosol [7]. Peroxynitrite (PN) is a widespread endogenous RNI, assembled spontaneously upon the interaction of nitric oxide (NO) with superoxide [8]. PN efficiently inactivates bacteria in cell-free systems [9,10], co-cultures consisting of professional phagocyte cells and pathogens [11], as well as in vivo [12,13]. A broad range of bacteria was shown to be vulnerable to inactivation by PN, i.e., *H. pylori* [10], *E. coli* [14], and *S. typhimurium* [15]. Furthermore, interference with the effects of PN augments hosted susceptibility to pathogens [9,16,17,18,19]. PN exerts its bactericidal effect via the oxidation of a broad range of biological molecules. The interaction of PN with proteins induces structural/chemical modifications, such as the oxidation of tryptophan, tyrosine, and cysteine residues, as well as the formation of carbonyl, dityrosine, and nitrotyrosine (NT) [20]. The presence of NT serves as a fingerprint for PN activity, both in vitro and in vivo, in various samples representing solid tissues, blood, and other clinically relevant homogenates. [21].

Most notably, an important property of PN is its ability to enhance innate and humoral immune responses against peptides or proteins, owing to NT formation. Cell-mediated immune responses were shown to be dramatically improved via the incorporation of NT instead of tyrosine into peptides, allegedly owing to better recognition by T cells [22,23,24]. Additionally, animals sensitized with NT-containing proteins exhibited higher antibody titers, directly proportional to the NT content of the protein. The antibodies generated against the modified proteins maintained their antigen specificity, exhibiting cross-reactivity with the unmodified proteins [25,26]. The study described in the current report further addresses the applicability of NT-mediated immunity improvement, in the case of a whole bacterial pathogen rather than individual proteinous antigens. This proof-of-concept study, employing the intracellular human pathogen *F. tularensis,* was conducted to assess the applicability of PN-induced inactivation protocols for developing vaccines, based on the administration of whole inactivated pathogens.

*F. tularensis*, the causative agent of tularemia, is a highly infectious, intracellular, Gram-negative bacterium. It is distinguished by two main subspecies that are pathogenic for humans. *F. tularensis* subsp. *tularensis* (biovar type A, a category A biothreat, Tier 1 Select Agent) is endemic in North America, causing a severe form of acute tularemia. *F. tularensis* subsp. *holarctica* (biovar Type B), found throughout Eurasia, exhibits attenuated virulence, causing a milder manifestation of the disease that is seldom fatal, and accordingly is classified as a category B biothreat agent [27,28]. *F. tularensis* is a facultative intracellular organism that infects a wide range of hosts and replicates within human and rodent macrophages. The host promiscuity of *F. tularensis* infections enabled the establishment of valuable models of tularemia in experimental animals, in particular the murine strains, which are instrumental in the study of the pathogenesis of the disease and the development of countermeasures. Following infection, *F. tularensis* employs strategies for immune evasion that delay the host immune response, resulting in enhanced morbidity [29]. For example, an important immune evasion mechanism relies on the fact that *F. tularensis* lipopolysaccharide (FT-LPS), an essential pathogen factor and potential immunogen, is unique in structure and consequently is not recognized by the host toll-like receptor-4 (TLR4) [30].

*F. tularensis* LVS is a live vaccine strain (LVS) derived from *F. tularensis* biovar type B. This strain, characterized by attenuated virulence in humans, is pathogenic in mice and is therefore commonly used to study this organism in rodent animal models. LVS is not a licensed vaccine in humans, mainly due to its residual reactogenicity and limited protective role against respiratory challenges [31]. Attempts to develop vaccines based on inactivated bacteria showed limited efficiency. Specifically, the inactivation of LVS with formalin elicited modest immune responses and consequently provided insufficient protection [30,31].

In the present study, pathogen inactivation by PN, combined with increased immunity of NT containing peptides, establishes a novel approach to *F. tularensis* vaccine development. PN-inactivated LVS formulations rapidly stimulate innate and adaptive immune cells, conferring efficient immune protection in a murine model of tularemia, superior to formalin-based formulations. This *F. tularensis* feasibility study suggests that the approach may be implemented for other pathogens.

## 2. Materials and Methods

### 2.1. Bacteria

*F. tularensis* subsp. *holartica* strain LVS (ATCC 29684) glycerol stocks were stored at −80 °C and streaked onto cysteine heart agar (CHA) (Becton Dickinson, Heidelberg, Germany) and incubated for 1–2 days at 37 °C. Bacterial cultures were grown at 37 °C to mid-log phase (optical density of 0.1–0.2 at 660 nm) in TSBC (TSB Difco, supplemented with 0.1% cysteine), washed and re-suspended in PBS, and used for either neutralization or infection, as described below.

### 2.2. Peroxynitrite-Induced Bacterial Inactivation

*F. tularensis* LVS bacteria were grown to reach 5 × 10^9^ CFU/mL. The growing medium was replaced with 10 mL of 0.2 M phosphate buffer, and then PN was added for 15 min (5 mM, 10 mM, or twice with 5 mM). The preparation was then neutralized by a 30 s incubation with 0.3 M HCl, and centrifuged (10,000 rpm) for 40 min. The pellet was re-suspended with 10 mL of phosphate buffer, aliquoted, and stored at −20 °C until used. Inactivation was confirmed by incubating the preparation on a cysteine heart agar (CHA) plate.

### 2.3. Formalin-Induced Bacterial Inactivation

*F. tularensis* LVS bacteria were grown to reach 5 × 10^9^ CFU/mL. The bacteria were then inactivated with 0.45% formalin for 24 h, washed twice with PBS, and re-suspended in phosphate buffer. The pellet was aliquoted and stored at −20 °C until used. Inactivation was confirmed by plating on a CHA plate.

### 2.4. Reduction of Nitrotyrosine Residues of the Peroxynitrite-Inactivated LVS Preparation

The PN-treated LVS preparation was centrifuged (10,000 rpm, 10 min) and re-suspended with 50 mM Tris-HCl, which was diluted in saline to reach pH-9. The NT residues were reduced to aminotyrosine by incubation with dithionite (final concentration of 25 mM) for 3 days, pelleted by centrifugation (10,000 rpm, 10 min), washed 3 times with PBS, and finally resuspended with PBS for a final concentration of 5 × 10^9^ CFU/mL.

### 2.5. Western Blot Analysis

Bacteria (2.5 × 10^9^ CFU/mL) were pelleted and suspended in 100 µL of PBS. Sample buffer (BioRad, Hercules, CA, USA) was added, and the samples were boiled for 5 min at 100 °C. Samples were resolved by 4–12% acrylamide gradient gel (30 µL/lane) and transferred onto nitrocellulose membranes. Membranes were blocked with 5% milk in Tris-buffered saline/Tween (TBST) for 1 h at room temperature. For the immunoblotting of NT residues, the membrane was incubated with anti-NT monoclonal antibody (clone 1A6, Millipore, 05-233, Burlington, MA, USA) overnight (1:1000 in 5% milk/TBST). The blot was extensively rinsed with PBS and incubated with anti-mouse horseradish peroxidase-linked antibody (1:5000 in 2.5% milk/TBST). The blot was extensively washed with TBST and developed with a homemade ECL reagent. The blot was visualized by a chemiluminescence detection system (Fujifilm, LAS3000, Tokyo, Japan).

### 2.6. Cells

*J774* (ATCC-TIB-67) were cultured in Dulbecco’s modified Eagle medium (DMEM) with 10% heat-inactivated fetal bovine serum (FBS), 10 mM non-essential amino acids (NEAA), 2 mM L-Glutamine, 1 mM sodium pyruvate, and 1% penicillin–streptomycin (all from Biological Industries, Beit Haemek, Israel) at 37 °C, 5% CO_2_. *MH-S* (ATCC-CRL-2019) were cultured in RPMI-1640 with 10% heat-inactivated fetal bovine serum (FBS), 10 mM non-essential amino acids (NEAA), 2 mM L-Glutamine, 1 mM sodium pyruvate, and 1% penicillin–streptomycin.

*U937* (ATCC-CRL-1593.2) were cultured in RPMI-1640 with 10% heat-inactivated fetal bovine serum (FBS), 10 mM non-essential amino acids (NEAA), 2 mM L-glutamine, 1 mM sodium pyruvate, and 1% penicillin/streptomycin, and were pulsed 72 h prior to the experiment with 100 ng/mL of phorbol myristate acetate (PMA) (Sigma-Aldrich, Israel) to induce macrophage differentiation at 37 °C, 5% CO_2_ (unless stated otherwise, all reagents are from Biological Industries, Israel). All cell lines tested negative for mycoplasma.

*Bone-marrow-derived dendritic cells (BMDC) preparation*. BMDCs were prepared essentially according to the method developed by Lutz et al. [32]. Briefly, bone marrow cells were flushed from femurs of 6- to 10-week-old male mice. Cells were passed through a cell restrainer, washed in phosphate-buffered saline (PBS), and re-suspended in a medium containing 50 ng/mL of recombinant murine granulocyte–macrophage colony-stimulating factor (rmGM-CSF) (R&D Systems) to a concentration of 1 × 10^6^ to 1.5 × 10^6^ cells/mL. Next, 10 mL aliquots of cell suspension were seeded into 100 mm Petri dishes (Falcon 351029). After 3 days, an additional 10 mL of fresh medium containing 40 ng/mL rmGM-CSF was added. Three days later, half of the culture supernatant was collected by centrifugation, and the cell pellet was resuspended in 10 mL of fresh medium containing 20 ng/mL of rmGM-CSF and added back to the culture. At day 8, nonadherent and slightly adherent cells were harvested by pooling cell supernatant and collecting cells by a gentle wash. Cells were centrifuged, re-suspended in a fresh medium containing 10 ng/mL of rmGM-CSF but lacking antibiotics, and seeded onto 100 mm cell culture dishes (Falcon 353003). Nonadherent cells were collected on day 9 and concentrated by centrifugation and resuspended in growth medium containing 10 mM HEPES buffer to a suspension of 10^6^ cells/mL to be used in the various experiments. More than 90% of the cells were CD11c-positive, as assessed by flow cytometry, indicating effective differentiation into DC. Light microscopy revealed the characteristic dendritic morphology, as well as cell clustering. DCs were grown in RPMI 1640 medium supplemented with 10% FCS, 2 mM l-glutamine, 1 mM sodium pyruvate, 1% (*vol*/*vol*) MEM-Eagle non-essential amino acid solution, 100 U/mL of penicillin, 100 μg/mL of streptomycin, and 50 μM β-mercaptoethanol (all components supplied by Biological Industries, Beit Haemek, Israel).

### 2.7. Cytokine Analysis

The TNFα concentrations were quantified by an ELISA kit (DuoSet, R&D BioSystems, Minneapolis, MN, USA) according to the manufacturer’s protocol.

### 2.8. Flow Cytometry

Primary dendritic cells were stained for the maturation markers CD40 (clone 1C10), CD54 (clone YN1/1.7.4), CD83 (clone Michel-17), CD86 (clone GL1), and MHC II (clone M5/114.15.2) for 20 min in flow buffer (PBS with 1% FCS and 0.05% sodium azide). Cells were analyzed on a FACSCalibur cytometer (Becton Dickinson, Franklin Lakes, NJ, USA), using the Cell Quest Pro software, and analyzed with FlowJo (Flowjo LLC, Ashland, OG, USA).

### 2.9. ELISPOT Assays

Mice were immunized with live LVS (the immunization protocol is described in the next paragraph). Spleens of immunized mice were dissociated in GentleMACS C-tubes (Miltenyi Biotec, Bergisch Gladbach, Germany), filtered and separated on lympholyte-M media according to the manufacture protocol. Single-cell suspensions were seeded into 96-well ELISPOT plates (MAIPS4510, Merck Millipore, Ireland) in complete RPMI medium, supplemented with 10% heat-inactivated fetal calf serum, 1 mM Pen-Strep, nonessential amino acids, 2 mM l-glutamine, 1 mM sodium pyruvate, 25 mM HEPES, and 50 µM β-mercaptoethanol. Neutralized *F. tularensis* LVS bacteria were used as stimulating antigens at a concentration of 5 × 10^7^ CFU equivalent per ml. Cells were plated for overnight incubation at a concentration of 10^6^/well and serially diluted to enable single-spot enumeration. Each sample was tested in duplicate. IFNγ-secreting cells were enumerated (Mouse IFNγ ELISPOT Ready-SET-Go!, eBioscience, San Diego, CA, USA) according to the manufacturer’s protocol.

### 2.10. Mice

All the animal experiments were approved by the IIBR committee for animal research. The experimental animals were handled according to the National Research Council 1996 Guide for the care and Use of Laboratory Animals and regulations of the IIBR Animal Use Committee. The IIBR animal experiment protocols were: M-24-2010, M-07-2011, M-37-2011, and M-49-2011. All mice were obtained from Charles Rivers. The mice were allowed free access to water and a rodent diet (Envigo, Israel). For the differentiation of bone-marrow-derived dendritic cells (BMDCs), 6 week-old female BALB/c mice were used. For the immunization experiments, female BALB/c mice (8–12 weeks old) were obtained. For the T cell response analysis, mice were anesthetized with ketamine and xylazine and inoculated intranasally with 10^2^ CFU in a volume of 25 µL. Five weeks later, mice were sacrificed, and splenocytes were collected for analysis. For the challenge experiments, mice were anesthetized as described above and given neutralized bacteria at a dose of 5 × 10^8^ CFU equivalent in a volume of 25 µL via the intranasal route. Fourteen days post-immunization, anesthetized mice were challenged with 1 × 10^4^ LVS *F. tularensis* bacteria intranasally.

### 2.11. Statistical Analysis

For all in vitro experiments, a two-way ANOVA analysis was performed. For in vivo experiments, a log-rank test was performed. All analyses were performed using GraphPad Prism version 5.01 for windows (GraphPad Software, San Diego, CA, USA).

## 3. Results and Discussion

### 3.1. PN Treatment Results in NT Formation and Inactivation of F. tularensis Bacteria

The present study aimed to explore the potential of PN-neutralized bacteria to serve in the development of a vaccine platform by exhibiting high antigenicity and immunogenicity, and consequently elicit a strong protective response. The study was conducted with the pathogen *F. tularensis* (FT), which is known to require the activation of a variety of immune mechanisms for protective immunity [28].

**Figure 1:**
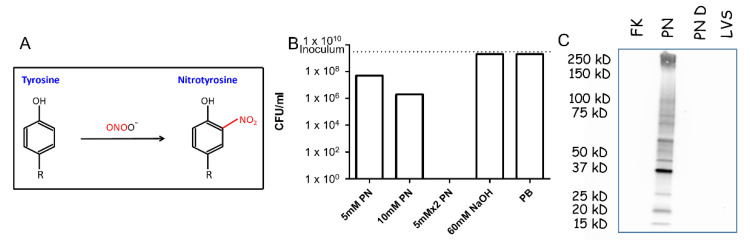
Peroxynitrite treatment of *F. tularensis*. (**A**) Modification of tyrosine residues by peroxynitrite (PN): the tyrosyl (phenol) group is nitrated, generating nitrotyrosine. (**B**) PN inactivation of *F. tularensis*: 5 × 10^9^ CFU/mL *F. tularensis* was incubated with 5 mM or 10 mM PN for 15 min, or twice with 5 mM PN. As a control, bacteria were incubated with 60 mM of NaOH. The LOD for viable bacterial counts is 5 CFU/mL. (**C**) Western blot analysis of inactivated LVS cells. Lysates of inactivated LVS by formalin (formalin-killed, FK), peroxynitrite (PN), peroxynitrite-inactivated bacteria treated with dithionite (PN D), and untreated bacteria (LVS) were analyzed by a specific anti-NT antibody. Equal amounts (10^8^ cell equivalents) of protein were loaded in each lane.

One of the most characteristic modifications induced by PN in polypeptides involves converting tyrosine residues to NT, resulting from the PN-mediated reaction of the phenol group (see Figure 1A). This modification represents a hallmark of PN activity and serves as a criterion for optimization experiments determining the FT cell-inactivating efficacy by PN (Figure 1B,C). Treatment of the bacteria with 5 mM or 10 mM PN for 15 min resulted in incomplete inactivation as evaluated by viable counting of colonies generated by plating bacteria following the PN treatment. However, when bacteria were treated twice with 5 mM PN, complete neutralization (more than nine orders of magnitude, Figure 1B) was observed. The need for the double treatment for achieving full neutralization may be due to the very short half-life of PN in the inactivation reaction.

In order to confirm the PN-mediated protein modifications (NT formation), the inactivated bacteria were analyzed by Western blot probed with anti-NT monoclonal antibodies (mAb). NT residues were detected only in the lysate of PN-inactivated bacteria, while no signals were detected in formaldehyde-neutralized bacteria or live bacteria. Of note, the presence of NT residues was verified by treatment of PN-inactivated bacteria with dithionite, an agent which reduces NT to aminotyrosine. Indeed, this treatment resulted in the complete abrogation of the anti-NT mAb recognition of bacterial proteins (Figure 1C).

### 3.2. Activation of Innate Immune Mechanisms by PN-Neutralized Whole Bacteria

**Figure 2:**
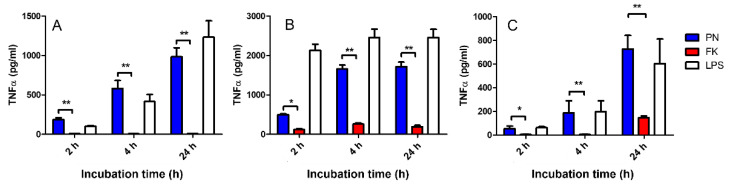
Macrophage response to whole inactivated bacteria. J774 cells (**A**), U937 cells (**B**), and MH-s cells (**C**) were incubated with formalin (FK)- or peroxynitrite (PN)-neutralized *F. tularensis* LVS bacteria. Macrophage activation was quantified by TNFα secretion into the media. *E. coli* LPS (LPS) was used as a positive control; (*) *p* < 0.05, (**) *p* < 0.01.

Next, the potential of PN-neutralized bacteria to induce an innate immune response was evaluated in vitro using a macrophage activation assay. Formalin- and PN-neutralized bacteria were co-incubated with three different macrophage cell lines: human U937 cell line, mouse J774, and mouse alveolar macrophage MH lines. They were then compared to LPS as a positive control. The cellular response was evaluated by monitoring TNFα secretion (Figure 2). In this set of experiments, TNFα served as a readout for an early, innate, pro-inflammatory response, together with IL-1β and IL-6, which are all centrally regulated by NF-κB [33]. A comparable pattern of activation was observed for all the examined cell lines. While PN-neutralized bacteria induced TNFα secretions after only two hours of incubation in all cell lines and time points, induction by formalin-inactivated bacteria required 4–24 h, and it was not observed in all cell lines.

We therefore conclude that PN-neutralized antigens are superior to formalin-neutralized antigens for macrophage activation.

**Figure 3:**
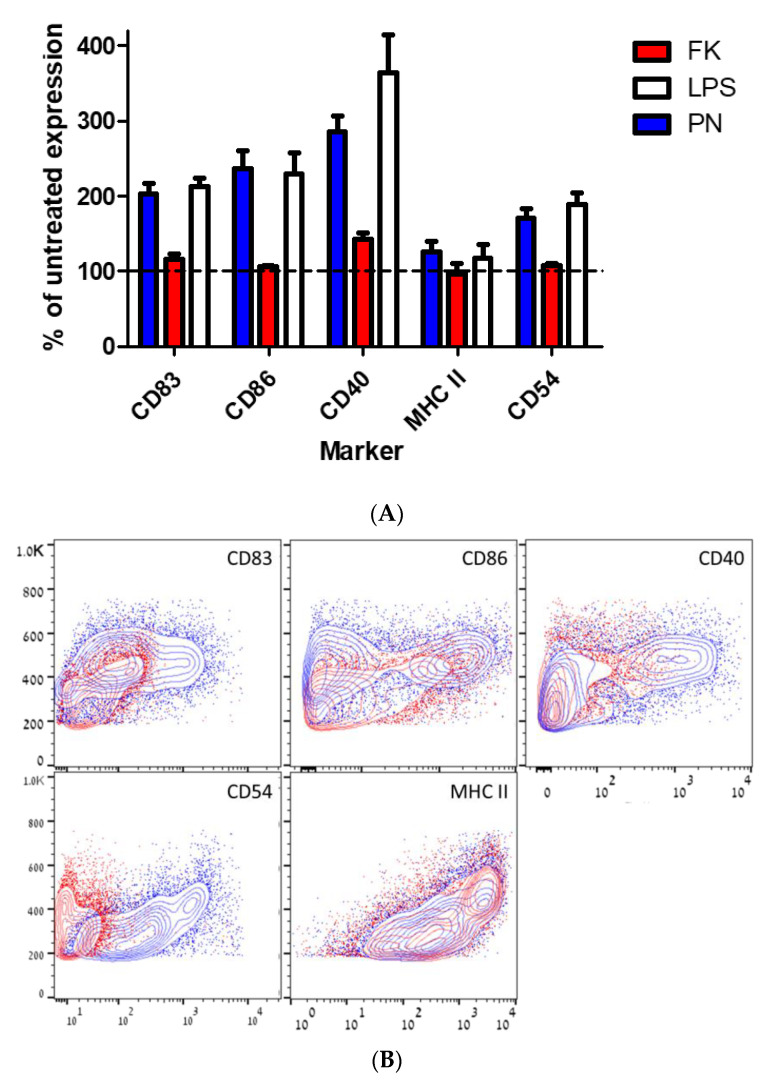
Early dendritic cell (DC) maturation, following incubation with neutralized bacteria. BMDCs were incubated for four hours with formalin (FK) or PN-neutralized bacteria, as indicated in the inset legend, stained for maturation markers expression and analyzed by flow cytometry. (**A**) The data are presented as the percentage of mean fluorescence intensity (MFI) change compared to non-treated cells. LPS (100 ng/mL) served as a positive control for DC maturation. (**B**) Representative dot blot presentation of CD11c^+^ cells following 24 h of incubation with FK- (red) or PN- (blue) inactivated bacteria presenting each marker (horizontal axis) versus forward scatter.

Dendritic cells (DCs) are the most potent T-cell inductive antigen-presenting cells of the immune system. One of the essential aspects in mounting an immune response is the DC maturation process, manifested by functional and phenotypic changes, which may serve as a reliable criterion for the evaluation of efficient activation. In order to examine their potential to induce DC maturation, neutralized bacteria were co-incubated for 4 h with primary, bone-marrow-derived DCs (BMDC). BMDCs were then evaluated for the expression of the maturation markers CD40, CD54, CD83, and CD86, as well as class II MHC (Figure 3). Compared to the expression level of non-treated cells, PN-neutralized bacteria induced the expression of all four maturation markers examined, while only a marginal effect was detected when formalin-inactivated bacteria were used in the assay. In both cases, the induction of MHC II was limited.

Therefore, the data obtained in the macrophage and DC activation assays strongly support the conclusion that PN-neutralized bacteria are highly potent inducers of innate immune responses, superior to formalin-inactivated bacteria.

### 3.3. Antigenicity Assessment of PN-Neutralized F. tularensis by ELISPOT

**Figure 4:**
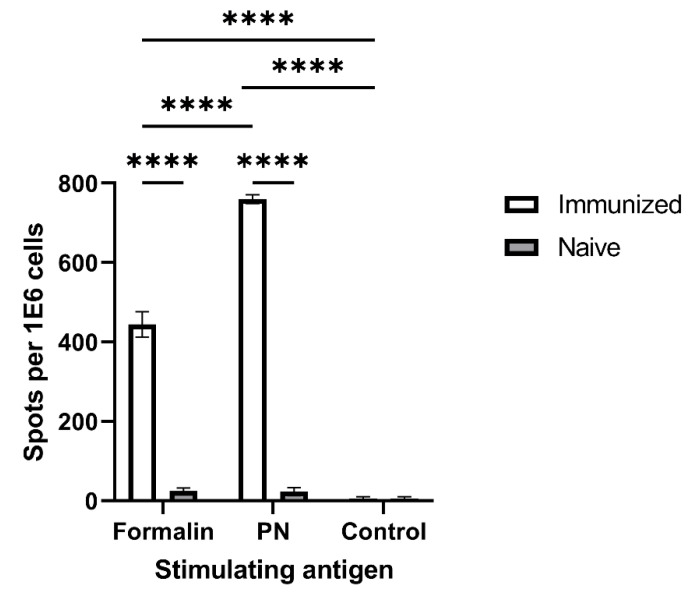
T cell response to whole bacterial antigens inactivated by formalin or peroxynitrite. Splenocytes from immunized mice were incubated with formalin- or peroxynitrite (PN)-neutralized *F. tularensis* LVS bacteria for 24 h. T cell response was measured by an IFNγ ELISPOT test. (****) *p* < 0.0001.

The antigenicity of neutralized *F. tularensis* bacteria was examined by quanitfying specific adaptive cellular responses against PN-treated bacteria in LVS-vaccinated mice. Accordingly, mice were immunized with live FT LVS.Five weeks later, their T cell response was assessed in an IFNγ-secretion ELISPOT assay using PN- or formalin-neutralized LVS as stimulating antigens. In this experimental setup, stimulating antigens were taken up by antigen-presenting cells, processed, and presented to T cells. Responsive T cells were then monitored via the secretion of the cytokine IFNγ (Figure 4), and spots representing individual specific-antigen responsive cells were enumerated. PN-neutralized LVS elicited a significantly higher number of IFNγ-secreting cells than formalin-inactivated bacteria. As expected, a minimal response to both antigens was detected with cells collected from naïve non-immunized mice. These data strongly support the desired antigenic cross-reactivity between naturally neutralized bacteria in vivo in the course of the immune response to infection and PN-neutralized bacteria in vitro.

Taken together, the above results (Figure 2, Figure 3 and Figure 4) clearly suggest that PN-inactivated bacterial formulations are superior to formalin-inactivated bacterial in terms of immune activation in vitro. It should be mentioned that, in sharp contrast to PN-inactivated bacteria, the immune response (TNFα secretion) following the co-incubation of macrophages with PN-inactivated, dithionite-treated bacteria (PN D) was completely abolished in all three cell lines tested (data not shown), indicating a direct role for PN-treated molecules in macrophage activation.

### 3.4. In Vivo Assessment of Protective Immunity Following Vaccination with Neutralized Bacteria

**Figure 5:**
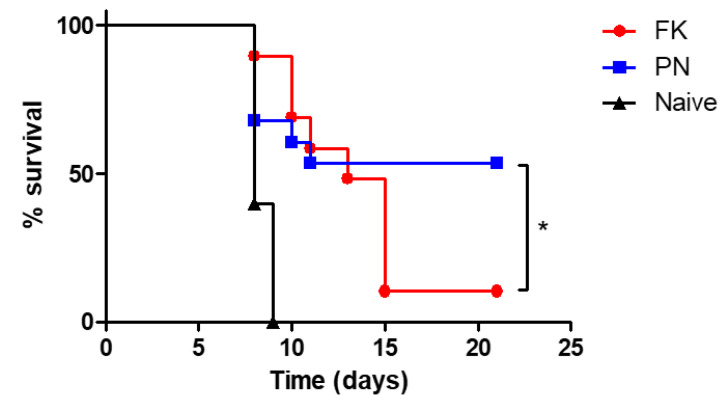
Survival of mice immunized by whole bacterial antigens inactivated by formalin (n = 14) or peroxynitrite (n = 14). Balb/c mice were immunized intranasal with 10^8^ cfu/mL of formalin (FK)- or peroxynitrite (PN)-inactivated bacteria. Fourteen days post-immunization, mice were challenged intranasal with 10 LD_50_ (10^4^ cfu/mL) of *F. tularensis* LVS. Survival was monitored for 21 days. Naïve mice (n = 10) were used as controls. (*) *p* < 0.05.

Given the superior potential of PN-neutralized bacteria to induce innate immune response as well as their high antigenicity, their potential to provide protective immunity against a lethal challenge with virulent bacteria was addressed. Mice were immunized twice with formalin- or PN-neutralized bacteria and, 14 days later, mice were intranasally challenged with a lethal dose of FT LVS bacteria (10^4^ CFU, equivalent to 10 x lethal dose 50% [LD_50_]). The data depicted in Figure 5 demonstrate that all naïve, non-immunized mice succumbed 8–9 days post-challenge. The immunization of mice with formalin-inactivated LVS did not induce significant protection following the LVS challenge (10% survival). In sharp contrast, a significant level of protection (50% survival) was promoted by immunization with PN-inactivated LVS. Our results are in accordance with previously described data, showing that vaccination with formalin-inactivated FT LVS is very limited in its ability to induce protective immunity [34]. That was also the case for additional FT models of vaccinations utilizing other chemically or heat-inactivated bacteria [34]. Vaccinations with live-attenuated FT strains are highly protective in animal models [35] and more potent than inactivated vaccines, owing to both differential gene expression under in vivo growth conditions and the presence of a live replicating organism. Interestingly, for immune-compromised people, limited in their potential to clear live bacteria, inactivated vaccines would be a better strategy.

This observation may imply that the addition of an adjuvant to PN-inactivated pathogen-based vaccines may be redundant, and thus unnecessary (or that a lower adjuvant concentration may be required), which may mitigate the probability of vaccine-induced adverse reactions.

## 4. Conclusions

The data presented in this report suggest that the inactivation of bacteria with PN may serve as a novel platform for vaccine preparation. This approach may be relevant for other strains of bacteria, as well as for viruses and toxins (a proof-of-concept was demonstrated in vitro for attenuated *Y. pestis* strain EV76, as well as for ricin toxin, data not shown). It should be emphasized that in the race to develop vaccines for SARS-CoV-2, the beta-propiolactone-inactivated whole virus was among the first vaccine platforms to be administered clinically (i.e., in China). However, these vaccines were proven ineffective, further emphasizing the importance of this work.

In conclusion, we presented a novel mode of pathogen neutralization by PN, superior to the commonly used formalin inactivation. The added value of retained antigenicity with adjuvanticity could be useful for future vaccine development.

## Data Availability

Not applicable.
